# Predictive value of novel inflammatory markers platelet-to-lymphocyte ratio, neutrophil-to-lymphocyte ratio, and monocyte-to-lymphocyte ratio in arterial stiffness in patients with diabetes: A propensity score–matched analysis

**DOI:** 10.3389/fendo.2022.1039700

**Published:** 2022-12-22

**Authors:** Peng Ning, Fan Yang, Jun Kang, Jing Yang, Jiaxing Zhang, Yi Tang, Yanghong Ou, Haiyan Wan, Hongyi Cao

**Affiliations:** Department of Endocrine and Metabolism, Geriatric Diseases Institute of Chengdu, Chengdu Fifth People’s Hospital (The Second Clinical Medical College, Affiliated Fifth People’s Hospital of Chengdu University of Traditional Chinese Medicine), Chengdu, China

**Keywords:** Platelet-to-lymphocyte ratio, neutrophil-to-lymphocyte ratio, monocyte-to-lymphocyte ratio, arterial stiffness, baPWV, diabetes

## Abstract

**Background:**

Increased arterial stiffness is common in patients with diabetes, and inflammation is one of the main causes of increased arterial stiffness. Platelet-to-lymphocyte ratio (PLR), neutrophil-to-lymphocyte ratio (NLR), and monocyte-to-lymphocyte ratio (MLR) are novel inflammatory markers that are reproducible, widely available, and easy to measure, and are associated with low costs. This study sought to investigate the predictive value of these novel inflammatory markers in patients with diabetes having arterial stiffness.

**Methods:**

We retrospectively included inpatients with diabetes mellitus from the Endocrinology Department of the Chengdu Fifth People’s Hospital from June 2021 to May 2022 and collected data on their general information, biochemical indicators, and brachial-ankle pulse wave velocity (baPWV). After propensity matching, the risk relationship between PLR, NLR, and MLR and arterial stiffness was assessed in the recruited patients.

**Results:**

A total of 882 hospitalized patients with diabetes were included in this study and categorized into the low baPWV (507 cases) or high baPWV group (375 cases) based on the baPWV. After propensity matching, there were 180 patients in all in the high and low baPWV groups. Univariate and multivariate logistic regression analyses revealed that high PLR, NLR, and MLR were independently associated with an increased risk of arterial stiffness in patients with diabetes. In the receiver operating characteristic curve analysis, the NLR area under the curve (AUC) was 0.7194 (sensitivity = 84.4%, specificity = 51.1%) when distinguishing low baPWV and high baPWV in patients with diabetes, which was higher than that for PLR AUC (0.6477) and MLR AUC (0.6479), and the combined diagnosis for AUC.

**Conclusions:**

NLR was superior to PLR, and MLR and combined diagnosis have certain predictive values that indicate the increase in arterial stiffness in patients with diabetes. These predictive values can help with the early identification of increased arterial stiffness in patients with diabetes.

## Introduction

Arterial stiffness is an important indicator of arterial elasticity and function. With an increase in age (1), the fibers degenerate and break, the content of collagen fibers increases, and the intima becomes thicker and stiffer, resulting in an increase in arterial stiffness and a decrease in compliance. Stiffness reflects changes in early vascular function and is considered to be closely related to cardiovascular and cerebrovascular events (2, 3). Patients with diabetes are more prone to increased arterial stiffness due to several complex metabolic disorders of sugars and lipids (4). Brachial-ankle pulse wave velocity (baPWV) is often used as an effective noninvasive index to assess peripheral arterial vascular stiffness owing to its reproducibility and simplicity (5), especially in patients with diabetes.

Vascular inflammation is a well-established risk factor and key pathogenic mediator in the development of endothelial dysfunction and consequent increase in arterial stiffness (6). Stimulated leukocytes may adhere and invade the vascular endothelium, resulting in capillary leukocyte arrest, vascular injury, and increased arterial stiffness (7, 8). Among leukocyte indices, the platelet-to-lymphocyte ratio (PLR), neutrophil-to-lymphocyte ratio (NLR), and monocyte-to-lymphocyte ratio (MLR) are the most suitable markers of vascular inflammation (9–11). These measurements offer distinct advantages over other established markers of inflammation owing to their excellent reproducibility, low assay costs, widespread availability, and simplicity of measurement (12). The ratio of these three distinct leukocyte subtype counts has evolved as a useful marker of vascular inflammation and has been found to correlate with arterial stiffness in many studies. However, there is still a lack of studies that have directly compared and evaluated which of PLR, NLR, MLR, and combined diagnosis is the better predictor of arterial stiffness. Therefore, in this study, we grouped patients with diabetes based on baPWV and investigated the association between PLR, NLR, and MLR and arterial stiffness.

## Patients and methods

### Study population

**Figure 1:**
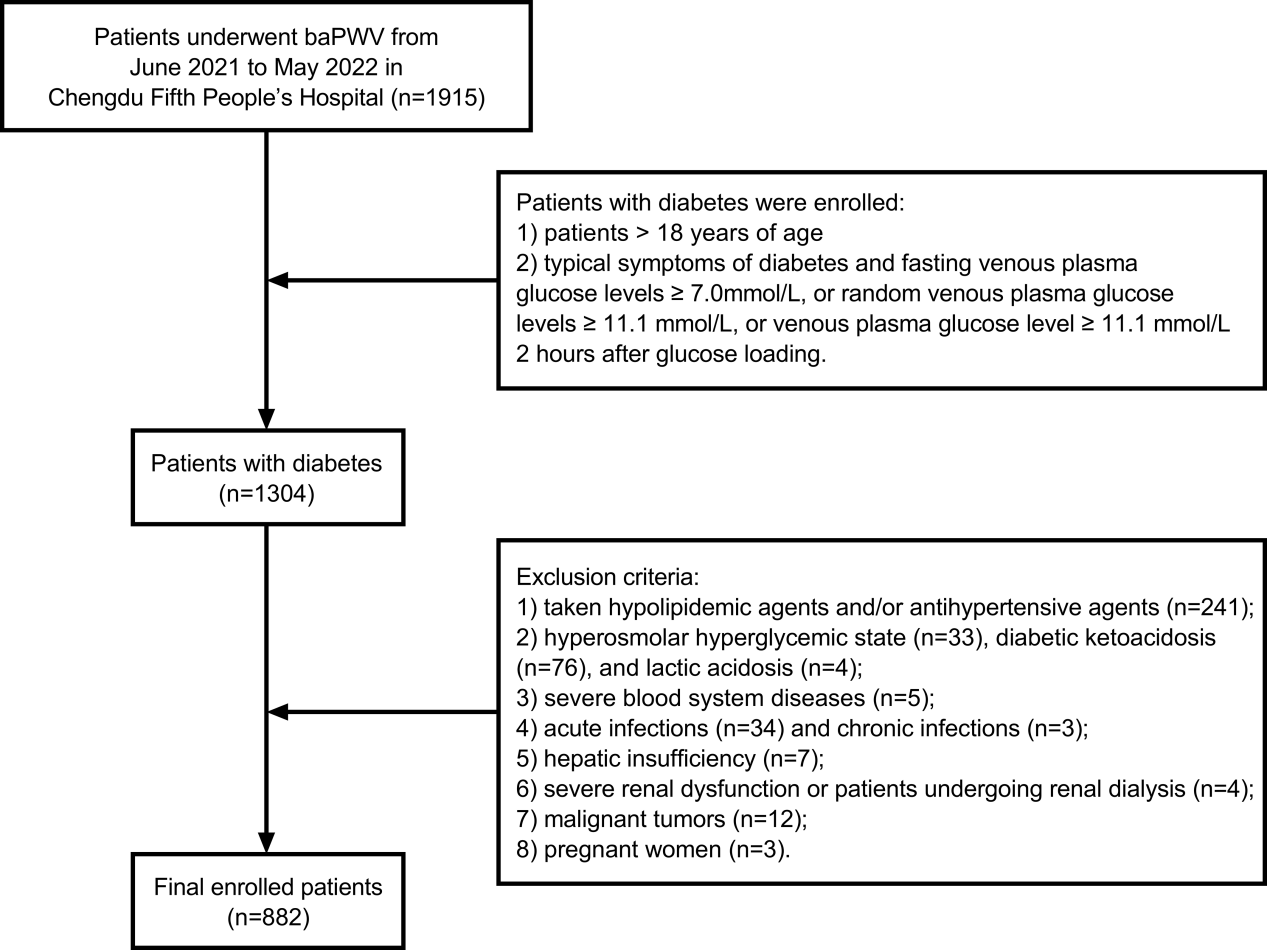
Flow chart of the study population enrollment.

A total of 882 patients with diabetes hospitalized in the Department of Endocrinology of the Chengdu Fifth People’s Hospital from June 2021 to May 2022 constituted the research subjects. The inclusion criterion was patients > 18 years of age who had a definite diagnosis of diabetes. Patients with symptoms of diabetes (polydipsia, polyuria, polyphagia, and unexplained weight loss) and fasting venous plasma glucose levels ≥ 7.0 mmol/L, random venous plasma glucose levels ≥ 11.1 mmol/L, or venous plasma glucose level ≥ 11.1 mmol/L 2 hours after glucose loading without the typical symptoms of diabetes were indicated blood glucose retesting on another day to confirm the diagnosis. The exclusion criteria were as follows: 1) patients taking hypolipidemic agents and/or antihypertensive agents in the past 6 months; 2) patients experiencing acute complications of diabetes, such as hyperosmolar hyperglycemic state, diabetic ketoacidosis, and/or lactic acidosis; 3) patients with severe blood system diseases; 4) patients with acute or chronic infections; 5) patients with hepatic insufficiency, and ALT and/or AST > 3 times the upper limit of normal; 6) patients with severe renal dysfunction, manifesting with an estimated glomerular filtration rate (eGFR) ≤ 15 mL/min × 1.73 m^2^ or patients undergoing renal dialysis; 7) patients with malignant tumors; 8) pregnant women (**Figure 1**). This study was conducted according to the principles outlined in the Declaration of Helsinki and was approved by the Medical Ethics Committee of the Chengdu Fifth People’s Hospital (No. 2022-020S-01).

### Measurements

The gender, age, duration of diabetes, smoking status, and drinking status of patients were collected by consulting their medical records. Blood pressure, height, and weight of patients were routinely measured, and general parameters, such as the body mass index (BMI), were calculated. baPWV was measured using the OMRON BP-203RPE III Arterial Stiffness Detection Device (Omron Co. LTD, Dalian, China) by professionally trained staff. Clinical data from our hospital laboratory were collected and analyzed using a Mindray BC-6800 Plus automatic blood cell analyzer (Mindray Biomedical Electronics Co., Ltd, Shenzhen, China) with reagents provided by Mindray to determine platelet, neutrophil, monocyte, and lymphocyte levels. A HITACHI LABOSPECT 008AS automatic biochemical analyzer (Hitachi Instruments Co., LTD, Shanghai, China) and reagents provided by HITACHI were used to measure fasting blood glucose (FBG), triglyceride (TG), total cholesterol (TC), high-density lipoprotein cholesterol (HDL-C), low-density lipoprotein cholesterol (LDL-C), and uric acid (UA) levels. Glycated hemoglobin (HbA1c) levels were determined using liquid chromatography. Lifotronic H9 hemoglobin detector (Lifotronic Technology Co., LTD, Shenzhen, China) reagent was provided by Lifotronic.

### Grouping criteria

Pulse wave velocity measurements are meaningful only if blood circulation is not disturbed during pulse propagation; therefore, it is important to determine whether arterial circulation in the lower extremities between the ilium and tibia is normal before measuring baPWV. When obtaining baPWV, we first measured the ankle-brachial index (ABI) of the ipsilateral limb. If the unilateral ABI is ≤ 0.9, it is necessary to first determine whether the limb is affected by peripheral vascular disease. In this case, the predictive value of baPWV will be doubted, so it was excluded. ABI > 0.9 on both sides is indicative of no severe obliterative arterial stiffness, whereas baPWV ≥ 1800 ms on either side is considered a better predictor of target organ damage (13–15). Therefore, we classified patients with baPWV ≥ 1800 ms into the high baPWV group and those with baPWV < 1800 ms into the low baPWV group.

### Statistical analysis

IBM SPSS 27.0 was used for data entry, processing, and statistical analysis, and GraphPad Prism 9.4.1 was used to construct calibration graphs, forest graphs, and receiver operating characteristic (ROC) curves. The baseline data of both groups were matched using a 1:1 ratio using propensity score matching, and the caliper value was set at 0.02. Independent samples *t*-test used to compare the two groups when the data were normally distributed. When the data showed skewed distribution, the Mann-Whitney U test of independent samples was used to compare both groups. Chi-square analysis was used to compare the categorical variable data between groups. The PLR, NLR, and MLR values were transformed into binary variables. Univariate and multivariate logistic regression analyses were conducted in succession. Goodness-of-fit tests were performed using the Hosmer-Lemeshow test and calibration plots were drawn. The ROC curve was used to analyze and evaluate the efficacy of PLR, NLR, MLR, and combined diagnosis for arterial stiffness. The corresponding area under the curve (AUC) was calculated, and the sensitivity and specificity corresponding to the maximum Youden’s index was calculated. Normally distributed data are presented as the mean ± standard deviation (SD), whereas data with a skewed distribution are presented as median (upper and lower quartiles) [M (QR)]. A P-value of < 0.05 was considered statistically significant.

## Results

### Baseline characteristics

A total of 882 patients with diabetes were enrolled in this study. General information, including the gender, age, duration of diabetes, height, weight, BMI, blood pressure, smoking status, drinking status of patients; and the biochemical indicators, including HBA1c, FBG, TG, TC, HDL-C, LDL-C, and UA, were determined (**Table 1**). Patients were divided into a low baPWV group (507 cases) or a high baPWV group (375 cases) based on their baPWV. The baseline data of the patients were matched using propensity score matching. There were significant differences in gender, age, disease course, DBP, SBP, HBA1c, and UA (all P < 0.05) before propensity score matching. After propensity scores matching by gender, age, disease course, BMI, SBP, DBP, smoking status, drinking status, and HBA1c, FBG, TG, TC, HDL-C, LDL-C, and UA levels, all parameters were well matched between both groups (all P > 0.05) (**Table 2**). Both before and after propensity score matching, we found that all patients in the high baPWV group had higher levels of PLR, NLR, and MLR than those in the low baPWV group (**Table 3**).

**Table 1:** Patients’ profiles.

Characteristics	All patients (n=882)
Gender (Male/Female)	507/375
Age (year)	58.69 ± 12.73
Course (year)	6.00 (1.00;10.00)
Height (cm)	161.56 ± 8.32
Weight (kg)	63.10 ± 11.78
BMI (kg/m^2^)	24.07 ± 3.48
SBP (mm Hg)	128.97 ± 20.12
DBP (mm Hg)	72.16 ± 12.94
Smoking (n/%)	322 (36.5)
Drinking (n/%)	294 (33.3)
HBA1c (%)	9.51 ± 2.66
FBG (mmol/L)	9.49 ± 4.35
TG (mmol/L)	2.59 ± 2.30
TC (mmol/L)	4.91 ± 1.33
HDL-C (mmol/L)	1.12 ± 0.34
LDL-C (mmol/L)	2.78 ± 0.97
UA (μmol/L)	337.18 ± 104.98
Platelets (×10^9^/L)	178.40 ± 60.50
Neutrophils (×10^9^/L)	4.18 ± 1.47
Monocytes (×10^9^/L)	0.36 ± 0.14
Lymphocytes(×10^9^/L)	1.53 ± 0.57
PLR	129.87 ± 62.86
NLR	3.14 ± 2.07
MLR	0.26 ± 0.14

**Table 2:** Patients’ profiles propensity score matching.

Variables	Before propensity matching			After propensity matching		
	Low baPWV n=520	High baPWV n=362	P-value	Low baPWV n=180	High baPWV n=180	P-value
Gender (Male/Female)	320/200	187/175	0.040	95/85	100/80	0.597
Age (year)	53.55 ± 11.30	66.06 ± 10.93	<0.001	61.86 ± 9.29	62.14 ± 10.87	0.790
Course (year)	4.00 (0.17;10.00)	10.00 (3.00;13.00)	<0.001	8.00 (1.00;12.00)	7.00 (2.00;10.00)	0.700
BMI (kg/m^2^)	24.21 ± 3.61	23.87 ± 3.28	0.154	24.14 ± 3.72	24.40 ± 3.14	0.473
SBP (mmHg)	121.53 ± 16.20	139.66 ± 20.40	<0.001	132.09 ± 17.19	132.02 ± 18.17	0.967
DBP (mmHg)	69.77 ± 10.59	75.59 ± 15.09	<0.001	72.26 ± 13.00	73.81 ± 14.281	0.282
Smoking (n/%)	199 (38.3)	123 (34.0)	0.193	64 (35.6)	68 (37.8)	0.662
Drinking (n/%)	179 (34.4)	115 (31.8)	0.411	58 (32.2)	59 (32.8)	0.910
HBA1c (%)	9.66 ± 2.72	9.30 ± 2.57	0.047	9.21 ± 2.71	9.02 ± 2.38	0.486
FBG (mmol/L)	9.60 ± 4.33	9.34 ± 4.37	0.384	9.18 ± 4.17	9.33 ± 4.15	0.730
TG (mmol/L)	2.66 ± 2.31	2.49 ± 2.29	0.286	2.30 ± 2.07	2.32 ± 2.16	0.937
TC (mmol/L)	4.95 ± 1.23	4.86 ± 1.48	0.374	4.82 ± 1.24	4.73 ± 1.31	0.466
HDL-C (mmol/L)	1.11 ± 0.34	1.14 ± 0.34	0.197	1.15 ± 0.33	1.13 ± 0.33	0.517
LDL-C (mmol/L)	2.82 ± 0.93	2.71 ± 1.04	0.101	2.69 ± 0.90	2.67 ± 1.01	0.792
UA (μmol/L)	325.80 ± 95.59	353.54 ± 115.35	<0.001	331.57 ± 92.10	338.14 ± 107.07	0.533

**Table 3:** Comparison of PLR, NLR, and MLR propensity scores before and after matching.

Variables	Before propensity matching			After propensity matching		
	Low baPWV n=520	High baPWV n=362	P-value	Low baPWV n=180	High baPWV n=180	P-value
PLR	119.55 ± 49.47	144.71 ± 75.84	<0.001	114.30 ± 51.99	150.69 ± 88.37	<0.001
NLR	2.66 ± 1.29	3.83 ± 2.70	<0.001	2.55 ± 1.59	3.93 ± 3.30	<0.001
PLR	0.23 ± 0.11	0.30 ± 0.16	<0.001	0.24 ± 0.12	0.30 ± 0.15	<0.001

### Logistic regression analysis to determine the relationship between PLR, NLR, and MLR and arterial stiffness

**Figure 2:**
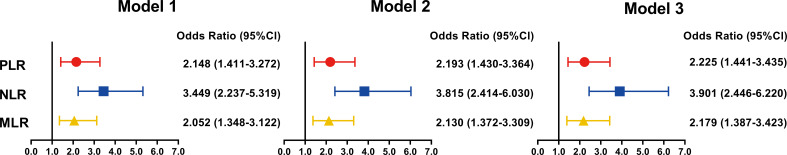
Univariate and multivariate logistic regression forest map of adjusted factors and the risk for arterial stiffness.

PLR, NLR, and MLR were converted into binary variables as independent variables, and Model 1 was constructed. Results from the univariate logistic regression analysis showed that high PLR, NLR, and MLR values were associated with the increased risk of arterial stiffness in patients with diabetes. Model 2 was constructed after adjusting for gender, age, disease course, BMI, smoking status, drinking status, SBP, and DBP. Model 3 was constructed after adjustment for HBA1c, FBG, TG, TC, HDL-C, LDL-C, and UA based on Model 2. Multivariate logistic regression analysis showed that PLR, NLR, and MLR were associated with an increased risk of arterial stiffness in patients with diabetes (**Table 4**). A forest plot was drawn (**Figure 2**).

**Table 4:** Logistic regression analysis of PLR, NLR, and MLR and the risk of arterial stiffness.

	Model 1				Model 2				Model 3			
Variables	B	S.E.	Wald	P-value	B	S.E.	Wald	P-value	B	S.E.	Wald	P-value
PLR	0.765	0.215	12.689	<0.001	0.785	0.218	12.950	<0.001	0.800	0.222	13.022	<0.001
NLR	1.238	0.221	31.385	<0.001	1.339	0.234	32.885	<0.001	1.361	0.238	32.686	<0.001
MLR	0.719	0.214	11.256	<0.001	0.756	0.225	11.329	<0.001	0.779	0.230	11.413	<0.001

Model 1: unadjusted.

Model 2: adjustment for gender, age, disease course, BMI, smoking, drinking, SBP, and DBP.

Model 3: adjustment for gender, age, disease course, BMI, smoking, drinking, SBP, DBP, HBA1c, FBG, TG, TC, HDL-C, LDL-C, and UA.

### PLR, NLR, and MLR clinically predicted arterial stiffness based on calibration diagram

**Figure 3:**
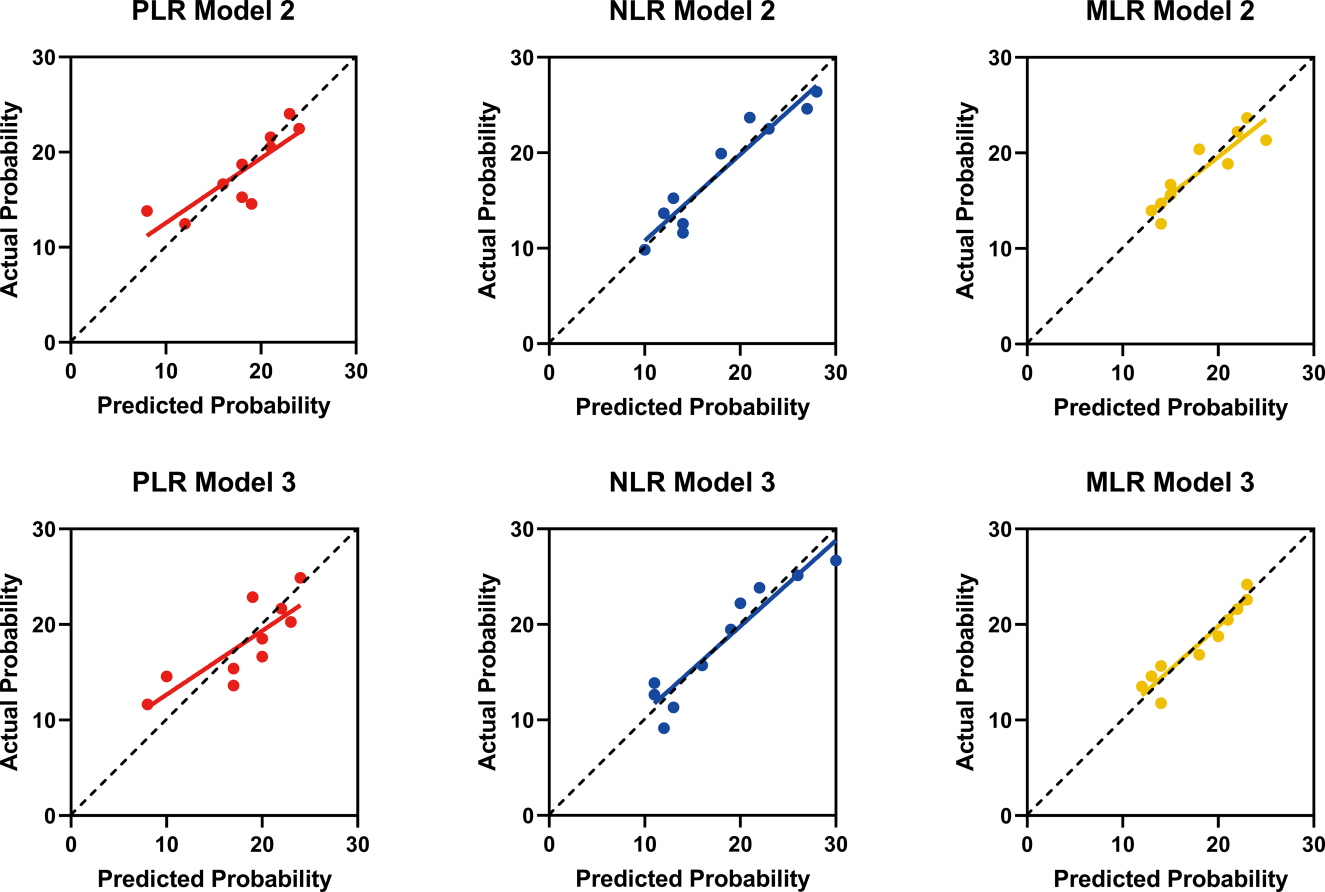
Calibration Diagram Model 2 and Model 3 of PLR, NLR, and MLR.

Actual and predicted probabilities were calculated using the multivariate logistic regression Models 2 and 3 of PLR, NLR, and MLR using the Hosmer-Lemeshow goodness-of-fit test. A calibration chart was drawn with the actual probability on the Y-axis as the dependent variable, and the predicted probability on the X-axis as the independent variable. The predicted probabilities of Models 2 and 3 of NLR and MLR are closer to the diagonal of the calibration plot (**Figure 3**).

### Predictive ability of PLR, NLR, and MLR in the diagnosis of arterial stiffness

**Figure 4:**
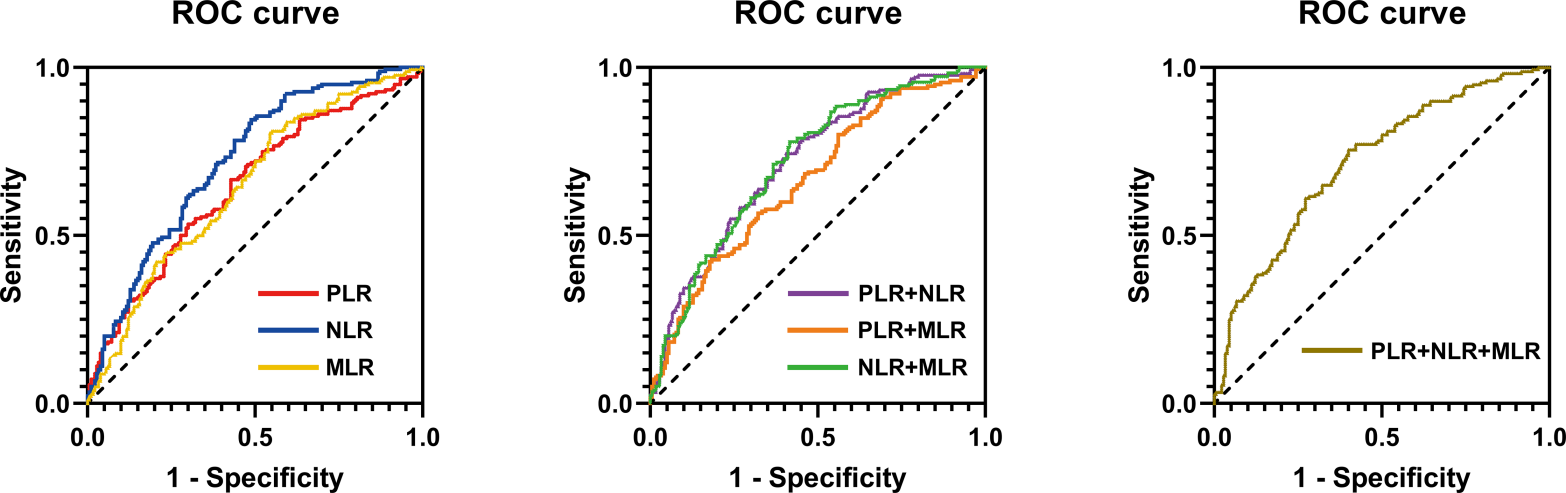
ROC curve for MLR, NLR, PLR, and combined diagnosis for predicting arterial stiffness.

The ability of PLR, NLR, and MLR in predicting arterial stiffness in patients with diabetes was further evaluated using high baPWV as a positive diagnostic result. PLR, NLR, MLR, PLR+NLR, PLR+MLR, NLR+MLR, and PLR+NLR+MLR values were used to construct the ROC curve and calculate the AUC. The AUC for NLR was 0.7194 (sensitivity = 84.4%, specificity = 51.1%), which was higher than that for PLR (AUC: 0.6477), MLR (AUC: 0.6479), PLR+NLR (AUC: 0.7185), PLR+MLR (AUC: 0.6646), NLR+MLR (AUC: 0.7177), PLR+NLR+MLR (AUC: 0.7192) (**Table 5** and **Figure 4**).

**Table 5:** ROC analysis of MLR, NLR, PLR, and combined diagnosis for arterial stiffness.

Variables	AUC	95% Cl	Youden’s index	Sensibility	Specificity	P-value
PLR	0.6477	0.591-0.704	0.239	66.7%	57.2%	<0.001
NLR	0.7194	0.667-0.772	0.355	84.4%	51.1%	<0.001
MLR	0.6479	0.591-0.704	0.262	80.6%	45.6%	<0.001
PLR+NLR	0.7185	0.666-0.771	0.338	77.4%	59.4%	<0.001
PLR+MLR	0.6646	0.609-0.720	0.245	42.8%	81.7%	<0.001
NLR+MLR	0.7177	0.665-0.770	0.361	77.8%	58.3%	<0.001
PLR+NLR+MLR	0.7192	0.667-0.772	0.356	75.6%	60.0%	<0.001

## Discussion

In this retrospective study, we first balanced the distribution of baseline variables in the low and high baPWV groups using propensity matching. After adjustment, the consistency of the baseline data between the two groups improved, thereby enhancing the comparability of the two groups of variables. Second, the levels of PLR, NLR, and MLR in the high baPWV group were higher than those in the low baPWV group. Next, we transformed the PLR, NLR, and MLR into binary variables and constructed Model 1 for univariate logistic regression analysis. After adjusting the general information and biochemical indicators of patients, Models 2 and 3 were constructed. Multivariate logistic regression analysis of factors revealed that high PLR, NLR, and MLR were independently associated with increased arterial stiffness in patients with diabetes, but the odds ratio for NLR was the highest, indicating the strongest correlation. We further calculated the actual and predicted probabilities of Models 2 and 3 of PLR, NLR, and MLR and found that the predicted probabilities of NLR and MLR were closer to the diagonal of the calibration diagram, indicating that Models 2 and 3 of NLR and MLR predicted the effect more accurately. Lastly, ROC curve analysis showed that the NLR value for the diagnosis of increased arterial stiffness in patients with diabetes was superior to the PLR and MLR values. The combined AUCs of NLR with PLR and MLR did not significantly increase compared with the AUC of NLR alone, indicating that the combined diagnosis does not lead to a significant increase in the diagnostic power for arterial stiffness in patients with diabetes. Moreover, our comparisons revealed that NLR had the highest sensitivity in diagnosing increased arterial stiffness. Therefore, NLR is recommended as a better predictor of arterial stiffness in individuals with diabetes.

Previous studies have found that inflammatory markers may be related to arterial stiffness. The relationship between NLR and arterial stiffness, especially, is the most studied. Park et al. found that elevated NLR was independently associated with arterial stiffness in a healthy population (16). Yi et al. found a correlation between NLR and vascular stiffness in healthy males (17). Wang et al. compared healthy subjects and individuals with hypertension and found that elevated NLR was a valid predictor of hypertensive arterial stiffness (18). Li et al. found that NLR was independently related to arterial stiffness in patients with acute coronary syndrome (19). Cai et al. found an independent association between NLR and arterial stiffness while studying patients who underwent peritoneal dialysis (20). Yu et al. found that the NLR in individuals with osteoporosis was higher and positively linked with baPWV (21). Some studies have reported the relationship between PLR and MLR and arterial stiffness. In a study of patients with peritoneal dialysis, Chen et al. found an independent association between high PLR and arterial stiffness (22). Wang et al. reported that MLR may be a potential predictor of arterial stiffness, but they used the cardio-ankle vascular index as a measure of arterial stiffness (23). However, these studies lacked a direct comparison of the three leukocyte subtype ratios in predicting the pros and cons of arterial stiffness. To the best of our knowledge, our study is the first to directly compare PLR, NLR, MLR, and the combined diagnosis to determine the one that was superior as a predictive value in evaluating arterial stiffness. In addition, the above studies are all retrospective in nature; thus, there will inevitably be uneven baselines during comparisons between the experimental and control groups. Our study was the first to adopt the method of tendency matching to reduce bias and ensure that the baseline of the data was as even as possible.

We found that high values of PLR, NLR, and MLR were independent predictors of increased arterial stiffness in patients with diabetes. Neutrophils, monocytes, and lymphocytes are representative leukocyte subtypes, and the mechanism of their involvement in increased arterial stiffness may be related to vascular adhesion protein (VAP)-1, which not only functions as an adhesion molecule but also as an amine oxidase that is involved in the production of aldehydes and hydrogen peroxide. Owing to its semicarbazide-sensitive amine oxidase activity, it is implicated in vascular injury, as the breakdown of primary amines releases formaldehyde and methylglyoxal, which cause direct cytotoxic damage to endothelial cells (24). The primary relationship between VAP-1 and arterial stiffness is inflammation, which causes an imbalance in the production and degradation of collagen and elastin fibers (25). VAP-1 functions as an adhesion molecule in the transfer of lymphocytes, granulocytes, and monocytes from the blood and participates in blood vessel–wall rolling, adhesion, and migration (26). It is also linked to endothelial dysfunction and AGE synthesis, both of which contribute to arterial stiffness. Our findings further strengthen the current evidence supporting the potential use of lymphocyte biomarkers to assess cardiovascular and cerebrovascular risk in subjects with diabetes. These biomarkers are superior to other conventional markers owing to their low cost, ease of analysis, and rapid results. Single blood parameters are easily disturbed by concentration, overhydration, and blood sample–processing methods among other factors. As PLR, NLR, and PLR values are ratios, their values are relatively more stable compared with single blood parameters (27). Arterial stiffness is a chronic condition that lasts for several years. However, it is clinically silent and difficult to detect in its initial stages; thus, it is typically detected at a later stage. Therefore, this simple and effective approach could help in the early identification of arterial stiffness in patients with diabetes.

In this study, we adopted baPWV as a method to measure arterial stiffness in inpatients with diabetes. BaPWV is based on oscillometry and widely used in clinical practice, especially in Asian countries, owing to its advantages of simplicity, noninvasiveness, and reproducibility (5). At the same time, arterial stiffness can also be reflected by measuring the PWV of other segments, such as the carotid-femoral PWV (cfPWV), carotid-radial PWV (crPWV), and aortic PWV (aPWV). However, baPWV and cfPWV are most widely used in clinical practice. Although cfPWV is the gold standard for measuring arterial stiffness based on arterial tonometry, they have a different focus than baPWV; cfPWV mainly reflects aortic stiffness, whereas baPWV mainly reflects peripheral arterial stiffness, which is not negligible in patients with diabetes. Meanwhile, baPWV is easier to operate compared with cfPWV. The blood pressure cuff needs to be bound to the limb of the patient without exposing the groin area. BaPWV is more suitable for epidemiological studies involving large populations and its reliability has been clinically verified (28). In addition to arterial tonometry and oscillometry, the other modalities for PWV measurement include ultrasound imaging (29), bioimpedance plethysmography (30), photoplethysmography (31), and magnetic resonance imaging (32). However, these methods are rarely used in clinical practice owing to limitations such as high price, complex operation protocols, and limited results. As this was a retrospective study, only baPWV was used to measure arterial stiffness. We intend to carry out prospective studies to increase our knowledge on cfPWV and other related indicators of arterial stiffness as a means to further explore the relationship between arterial stiffness and NLR, PLR, and MLR.

Our study has some limitations. This single-center study only included patients of Asian/Chinese descent. Whether the results of the study can be extrapolated to other races should be further determined. We used baPWV instead of the gold standard cfPWV as a measure of arterial stiffness. Although the American Heart Association recommends baPWV as a common measure of arterial stiffness (5), relative to cfPWV, baPWV may be limited by the inclusion of long arterial muscle segments. This may lead to misclassification of the assessment of arterial stiffness (33). As this was a retrospective study, we could not collect adequate data on inflammatory factors, such as hsCRP, IL-6, and TNF-α, which are associated with other chronic metabolic diseases. Unfortunately, these factors were not compared with PLR, NLR, and MLR.

## Conclusions

As a novel, convenient, and inexpensive inflammatory marker, NLR is superior to PLR, MLR, and combined diagnosis, and has a certain predictive value to identify the increase in arterial stiffness in patients with diabetes. Thus, this biomarker can be considered in a clinical setting to help identify the increase in arterial stiffness in individuals with diabetes as an approach to disease prevention or early intervention.

## Data availability statement

The original contributions presented in the study are included in the article/**Supplementary Material**. Further inquiries can be directed to the corresponding author.

## Ethics statement

The studies involving human participants were reviewed and approved by the Medical Ethics Committee of the Chengdu Fifth People’s Hospital(No. 2022-020S-01). Written informed consent for participation was not required for this study in accordance with the national legislation and the institutional requirements.

## Author contributions

PN and HC conceived and designed the study. JY, YT, and YO collected and cleared the data. PN and FY analyzed and interpreted the data. PN, JK, JZ, HW, and HC drafted and revised the manuscript. All authors contributed to the article and approved the submitted version.
